# Formulation and *in vitro* evaluation of meloxicam as a self-microemulsifying drug delivery system

**DOI:** 10.12688/f1000research.130749.2

**Published:** 2023-05-30

**Authors:** Saja Abdulkareem Muhammed, Khalid Kadhem Al-Kinani

**Affiliations:** 1 Department of Pharmaceutics, College of Pharmacy, University of Baghdad, Baghdad, Baghdad Governorate, Iraq

**Keywords:** Pseudo-ternary phase diagram, Dissolution rate, SMEDDS, Meloxicam

## Abstract

**Background:** The nonsteroidal anti-inflammatory medication meloxicam (MLX) belongs to the oxicam family and is used to reduce inflammation and pain. The aim of this study was to improve MLX's dispersibility and stability by producing it as a liquid self-microemulsifying drug delivery system since it is practically insoluble in water.

**Methods:** Five different formulations were made by adjusting the amounts of propylene glycol, Transcutol P, Tween 80, and oleic acid oil and establishing a pseudo-ternary diagram in ratios of 1:1, 1:2, 1:3, 1:4, and 3:4, respectively. All of the prepared formulations were tested for a variety of properties, including thermodynamic stability, polydispersity index, particle size distributions, dilution resistance, drug contents, dispersibility, *in vitro* solubility of the drug, and emulsification time.

**Results:** F5 was chosen as the optimal MLX liquid self-microemulsion due to its higher drug content (99.8%), greater *in vitro* release (100% at 40 min), smaller droplet size (63 nm), lower polydispersity index (PDI) value (0.3), and higher stability (a zeta potential of -81 mV).

**Conclusions:** According to the data provided here, the self-microemulsifying drug delivery system is the most practical method for improving the dispersibility and stability of MLX.

## Introduction

About half of all novel medication compounds are poorly soluble in water, and when given orally, they show little bioavailability. Solutions to these problems are presently being achieved *via* the use of a wide variety of formulation techniques, such as solid dispersion, cyclodextrin inclusion complexes, particle size reduction, oils, surfactants, penetration boosters, salt formulations, microparticles, and many more. Lipid solutions, emulsions, and emulsion pre-concentrates are physically stable formulations often employed for encapsulating poorly soluble medicines. ^1^

Self-micro emulsifying drug delivery systems (SMEDDS) are non-ionized, translucent, and thermodynamically stable systems. When injected into the aqueous phase with mild agitation, these systems spontaneously produce an oil/water microemulsion with globule diameters generally less than 200 nm, consisting of the co-surfactant, surfactant, oil, and medication. The agitation needed to generate microemulsions may be found in the digestive motility of the gastrointestinal system. ^2^ To increase the oral bioavailability of medications with limited water solubility, SMEDDS works to increase colloidal dispersibility and retain small oil globules containing the medication as it travels through the digestive tract. ^3^

Nonsteroidal anti-inflammatory medication (NSAID) meloxicam (MLX) works by blocking prostaglandin production, mainly the cyclooxygenase-2 (COX-2) isoform of cyclooxygenase, making it an effective treatment for pain and inflammation, fluid retention, and fever. When compared to other NSAIDs, it is shown to provide therapeutic advantages associated with osteoarthritis, rheumatoid arthritis, and other joint conditions. It is also showing great promise as a medication for treating diseases like Alzheimer's disease and cancer. However, MLX is only moderately soluble in aqueous solvents, leading to varying oral bioavailability and making it challenging to produce effective pharmaceutical formulations. Several techniques, including the creation of salts, have been tried to improve medication solubility. On the other hand, there is no guarantee that using a salt will be preferable to using the substance in its free form. Free MLX and its salt forms are both weakly soluble in aqueous systems at pH 4. MLX may produce sodium, potassium, and ammonium salts.

There are many delivery systems that can be used to formulate MLX, like nanoemulsion,microemulsion,soild dispersion, etc.

The microemulsion delivery system has the disadvantage of having a large amount of surfactants, stability is influenced by temperature, pH, and limited solubility capacity for substances with high melting points.

The nanoemulsion delivery system has the disadvantage of a large concentration of the surfactant and expensive techniques for preparation.

The disadvantages of solid dispersion are forming crystalline solid dispersion as they were prepared using crystalline carriers like urea and sugars. Crystalline solid dispersions were more thermodynamically stable, which lowered their dissolution rate as compared to amorphous ones.

While the SMEDDS deliver the drug with the easiest technique and faster drug release.

To overcome the low solubility of MLX, a self-emulsifying drug delivery system (SEDDS) might be used. Meloxicam is suitable for this system due to its poor water solubility, which causes low absorption,low dosage, no substantial first-pass metabolism, is highly soluble in the system ingredients, and has a high log p of 3.43. This study aimed to prepare and characterize MLX as a liquid (SMEDDS) to improve its stability, solubility, and colloidal dispersibility for appropriate MLX delivery through the oral cavity, since according to biopharmaceutical classification system (BCS) parameters, MLX belongs to class II, which is of low solubility and high permeability. ^4^

## Methods

This research was done at the Department of Pharmaceutics, College of Pharmacy, University of Baghdad, Baghdad, Iraq.

### Materials

MLX was obtained from Hyperchem (China), Transcutol p from Gattefosse Sas (France), Tween 80 and propylene glycol from Chemical Point (Germany), oleic acid oil from Central Drug House(P) LTD (India), methanol from Sigma-Aldrich (Bljika), and hydrochloric acid from ReAgent Chemicals (UK).

### Saturation solubility studies

A 2 mL glass tube of the selected vehicle was spiked with a large quantity of MLX powder. After the mixture was sealed, it was sonicated for five minutes, and then subjected to shaking for 48 hours in a shaker water bath (Memmert, model number: D3006) at (25°C). The mixture was then centrifuged at 3,000 rpm for 20 minutes, filtered through a 0.45 μm membrane filter, and dilution of oils, surfactant, co-solvent, and co-surfactant with methanol as a solvent. Then, the drug concentrations were determined using spectrophotometry (Shimadzu 1650 pc-Japan, model number: UV mini 1240) at their respective max (355 nm). Solubility data were expressed as mean±SD. ^5^

### Construction of pseudo-ternary phase diagrams

Transcutol P, propylene glycol, Tween 80, and oleic acid oil were used as the co-surfactant, co-solvent, surfactant, and oil phase, respectively, to conduct hydrophilic-lipophilic balancing value and solubility investigations. As a means of establishing the relative amounts of SMEDDS components, a pseudo-ternary phase diagram was drawn using a water titration method. The ratio of surfactant to co-surfactant has been adjusted, and the resulting mixture is called Smix (1:1, 1:2, 1:3 1:4 and 3:4). The Smix:oil combination was titrated with distilled water under mild magnetic stirring until a stable, clear, and transparent system was produced, the point of shift from clear to unclear was recorded. Origin 2018 64Bit software ^6^ (free alternative, GnuPlot) was used to generate a ternary plot using the gathered data. ^7^

### Preparing MLX liquid SMEDDS

A series of liquid SMEDDS formulas were created ( Table 1) by combining oleic acid as the oil, Tween 80 as the surfactant, Transcutol P as the co-surfactant and propylene glycol as the cosolvent in the ratios (1:1, 1:2, 1:3, 1:4, and 3:4), while keeping the oil: Smix ratio constant (1:9). The SEDDS components, such surfactants and oils, poorly dissolve MLX. A concentrated basic solution of salts, such as tris (hydroxymethyl) aminomethane (Trizma), can be added to the SEDDS in order to establish a suitably basic environment to solubilize MLX. The ratio of base to water in a solution is 1:2 by weight. The pH measurements showed that the solution has a pH of 11.1. The SMEDDS of oleic acid was created by adding the following substances to the Trizma buffer (20%) in the specified order: oleic acid, Tween 80, propylene glycol, and Transcutol p. All calculations were done based on actual weight. Ingredients were combined in a beaker using a magnetic stirrer (Faithful, model number: SH-2) and heated at 60°C for 30 minutes in a water bath to obtain a clear solution. Next, 7.5 mg MLX was added to the mixture, and it was blended for an additional hour, resulting in a clear, yellow liquid. The formulations were then kept for 48 hours while being visually inspected for turbidity and phase separation before droplet size distribution tests were performed. ^8^

**Table 1.** Composition of the prepared meloxicam liquid SMEDDS (% w/w). SMEDDS, Self-micro emulsifying drug delivery system.

Formula – code	Smix ratio	Oil: Smix ratio	Oleic acid oil	Tween 80	Transcutol P, Propylene glycol (1:1)
**SMEDDS-1**	1:1	1:9	10	45	45
**SMEDDS-2**	1:2	1:9	10	30	60
**SMEDDS-3**	1:3	1:9	10	22.5	67.5
**SMEDDS-4**	1:4	1:9	10	18	72
**SMEDDS-5**	3:4	1:9	10	38.6	51.4

### *In vitro* evaluation of the prepared MLX liquid SMEDDS

***Thermodynamic stability studies***

Numerous thermodynamic stability tests (centrifugation, heating-cooling cycle, and freeze-thaw cycle) were done on all the prepared liquid SMEDDS formulations to determine the effect of centrifugation and temperature on the stability of self-microemulsions and to avoid selecting metastable formulations for further development and characterization. ^9^

*Centrifugation test*

All SMEDDS formulations were centrifuged in Centrifuge (Eppendorf, model number: Hettich EBA 20) at 3,500 rpm for 30 minutes and tested for phase separation, creaming, precipitation, and cracking. For the heating-cooling cycle, stable formulations were chosen. ^10^

*Heating-cooling cycle (H/C cycle)*

The H/C cycle was used to investigate the temperature-dependent stability of self-microemulsions. Six cycles were performed. The cycles were performed between refrigerator temperatures (5°C) and 45°C, with each temperature being held for at least 48 hours. Formulations that remained stable at these temperatures were exposed to a freeze-thaw cycle. ^11^

*Freeze-thaw cycle*

For all manufactured SMEDDS formulations, three freeze-thaw cycles between -21 and +25°C were performed, with storage at each temperature for at least 48 hours. Formulations that passed these thermodynamic stress tests were chosen for additional tests. ^12^

***Droplet size measurement and polydispersity index (PDI)***

In order to determine the mean droplet size and PDI, 0.5 mL SMEDDS was dissolved in 250 mL distilled water, and the mixture was gently stirred with a magnetic stirrer at 25°C. The droplet size and PDI were measured using a Malvern Zetasizer (Malvern Zen 2112), which analyses the variation in light scattering due to Brownian motion of the particles. The angle of incidence was 173°, and the temperature was 25°C, to measure light scattering. ^13^

***Robustness to dilution***

In two separate glass vials, the obtained SMEDDS formulations were diluted to 50-, 100-, 1,000-, and 3,000-fold with distilled water (D.W) and 0.1 N HCl. After 24 hours, the diluted microemulsion formulations were shaken and visually evaluated for any phase separation, droplet coalescence, or drug precipitation. ^14^

***Dispersibility tests and self-microemulsifying time***

The efficiency and self-microemulsifying time were to be determined using the USP dissolving equipment II (paddle type) (Faithful RC-6). A half milliliter of SMEDDS formulation was added to 500 mL D. W and gently agitated at 37°C with a stirring speed of 50 rpm. When a transparent homogeneous system was created, the *in vitro* effectiveness of the formulations was visually examined. ^15^ Utilizing a grading system, the time (in minutes) for full microemulsifying was determined, ^16^ as shown in Table 2.

**Table 2.** Classification of the SMEDDS formulation in accordance to comparative grades. SMEDDS, Self-micro emulsifying drug delivery system.

Grade	Time for self-microemulsifying	Appearance
1 ^st^(A)	Rapidly forming microemulsion (within 1 min)	Showing a bluish or clear appearance
2 ^nd^(B)	Quickly forming (<2 min)	Slightly less transparent emulsion that appears blue white
3 ^rd^(C)	<2 min	Bright white emulsion
4 ^th^(D)	Slow to emulsify (>3min)	White, greyish dull emulsion showing a slightly oily appearance
5 ^th^(E)	Slow to emulsify (>3min)	Exhibiting either poor or minimal emulsification with large oil globules present on the surface

***Determination of drug content***

The drug content in SMEDDS formulation was determined by UV/visible Spectrophotometer. A quantity of 0.4 g (equivalent to 7.5 mg MLX) of each formulation was accurately measured and diluted to 100 mL with methanol. The resultant solutions were then analyzed spectrophotometrically at its λ max (355 nm) in methanol using UV-Visible Spectrophotometer Shimadzu 1650 pc-Japan. ^17^

***In vitro dissolution study***

All prepared SMEDDS formulations (those with a size less than 200 nm) were tested for drug release *in vitro* using the USP dissolution apparatus-II (paddle method) and 0.1N HCl as the dissolution media (900 mL) at 37±0.5°C and 50 rpm. ^18^ Dialysis bag technique (8,000-14,000 Da) was used. The dialysis bags were washed with deionized water and soaked in 0.1N HCl overnight to equilibrate. ^19^ A dialysis bag was filled with 0.4 g SMEDDS containing MLX equivalent to one dose (7.5 mg) and 5 mL of the dissolution medium was withdrawn every 10 minutes for 60 minutes (10, 20, 30, 40, 50 and 60 min). To maintain sink conditions, the withdrawn samples were replaced with an equal volume of fresh medium (0.1 N HCl). UV/visible spectrophotometer analysis determined the amount of drug dissolved in the withdrawn samples. ^20^

### Selection of optimum MLX liquid self-microemulsion formula

According to the *in vitro* evaluation studies (droplet size measurement, PDI, *in vitro* dissolution study and drug content) the best MLX liquid SMEDDS formula was chosen.

***Zeta potential measurements***

Only the chosen liquid SMEDDS formulation was subjected to zeta potential measurements. A magnetic stirrer was used to gently mix 1 mL SMEDDS and 10 mL DW at 25°C in order to perform the measurement. Zeta potential was determined using a Malvern Zetasizer instrument. ^21^

***Atomic force microscopy (AFM)***

The mode used in this characterization was the contact mode, which was done when the AFM tip makes direct contact with the sample surface, and the surface profiles are created by either fixed altitude or static load operation. ^22^ The device used was NaioAFM Nanosurf Switzerland.

***Field emission scanning electron microscope (FESEM)***

The chosen liquid SMEDDS formula's morphology was evaluated using FESEM. A section of the microemulsion sample was analyzed using FESEM TESCAN MIRA3 FRENCH. The samples were inspected at a variety of magnifications, and the resulting photographs were uploaded to computers for further analysis. ^23^

***Transmission Electron Microscope (TEM)***

The geometry and morphology of the final liquid SMEDDS formulation were analyzed by TEM. The powder is mixed with ethyl alcohol, then ultrasonically dispersed for 10 minutes before a single drop of the solution is poured onto a copper mesh with an amorphous carbon coating. After the specimen dried, it was examined using TEM equipped with a titanium filament as the electron beam projector and an image sensor. ^24^ These photos were taken by TEM Philips EM208S-100 Kv.

### Statistical analysis

The experimental data were summarized as the mean standard deviation of three samples (in triplicate), and one-way analysis of variance (ANOVA) followed by post hoc Tukey’s test was performed at a significance level of P<0.05 to assess whether or not the changes in the applied parameters were statistically significant.

## Results and discussion

### Saturation solubility of MLX in different oils, surfactants and co-surfactants

To identify the most suitable solvents for MLX dissolution, a saturation solubility test was carried out. Researchers have looked at whether or not MLX is soluble in a wide range of co-surfactants, co-solvent, oils, and surfactants. The solubility of MLX in oleic acid oil exhibited the highest solubility ( Table 3). ^42^

**Table 3.** Saturation solubility values of meloxicam in different oils.

Oil	Solubility (mg/mL) mean±SD *	Oil	Solubility (mg/mL) mean±SD *
Clove oil	0.843±0.046	Triacetin oil	0.813±0.0035
Sunflower oil	0.334±0.004	Corn oil	0.268±0.0015
Peppermint oil	0.951±0.002	Lemon oil	0.404±0.005
Oleic acid oil	2.337±0.003	Grape seed oil	0.165±0.001
Olive oil	1.491±0.003	Sweet Almond oil	1.164±0.014
Cod liver oil	1.646±0.04	Capryol 90 oil	0.82±0.001
Cinnamon oil	1.524±0.031	IPM oil	0.213±0.005
Labrafil 1944M	0.563±0.006	Sesame oil	0.526±0.023
Orange oil	0.312±0.006	Lavender oil	0.2717±0.01
Castor oil	0.914±0.016	Linseed oil	1.0625±0.048
		Cotton seed oil	0.578±0.007

* SD standard deviation from mean, n=3.

As shown in Table 4, ^40^^,^^44^ the surfactant Tween 80, the co-surfactant Transcutol P, and the cosolvent propylene glycol exhibited the highest solubility for MLX and were therefore selected for the study.

**Table 4.** Saturation solubility of meloxicam in different surfactants and co-surfactants.

Surfactant	Solubility (mg/mL) mean±SD *	Co-surfactant	Solubility (mg/mL) mean±SD *
Span 20	0.556±0.004	Transcutol P	13.88±0.024
Span 80	0.653±0.003	PEG 600	7.813±0.016
Tween 20	4.8503±0.009	PEG 400	8.302±0.061
Tween 60	4.974±0.060	Propylene glycol	0.747±0.011
Tween 80	9.063±0.058	Glycerol	0.592±0.004
Cremophor EL	7.669±0.071	Ethanol	0.601±0.010

* SD standard deviation from mean, n=3.

### Pseudo-ternary phase diagram construction

**Figure 1.**
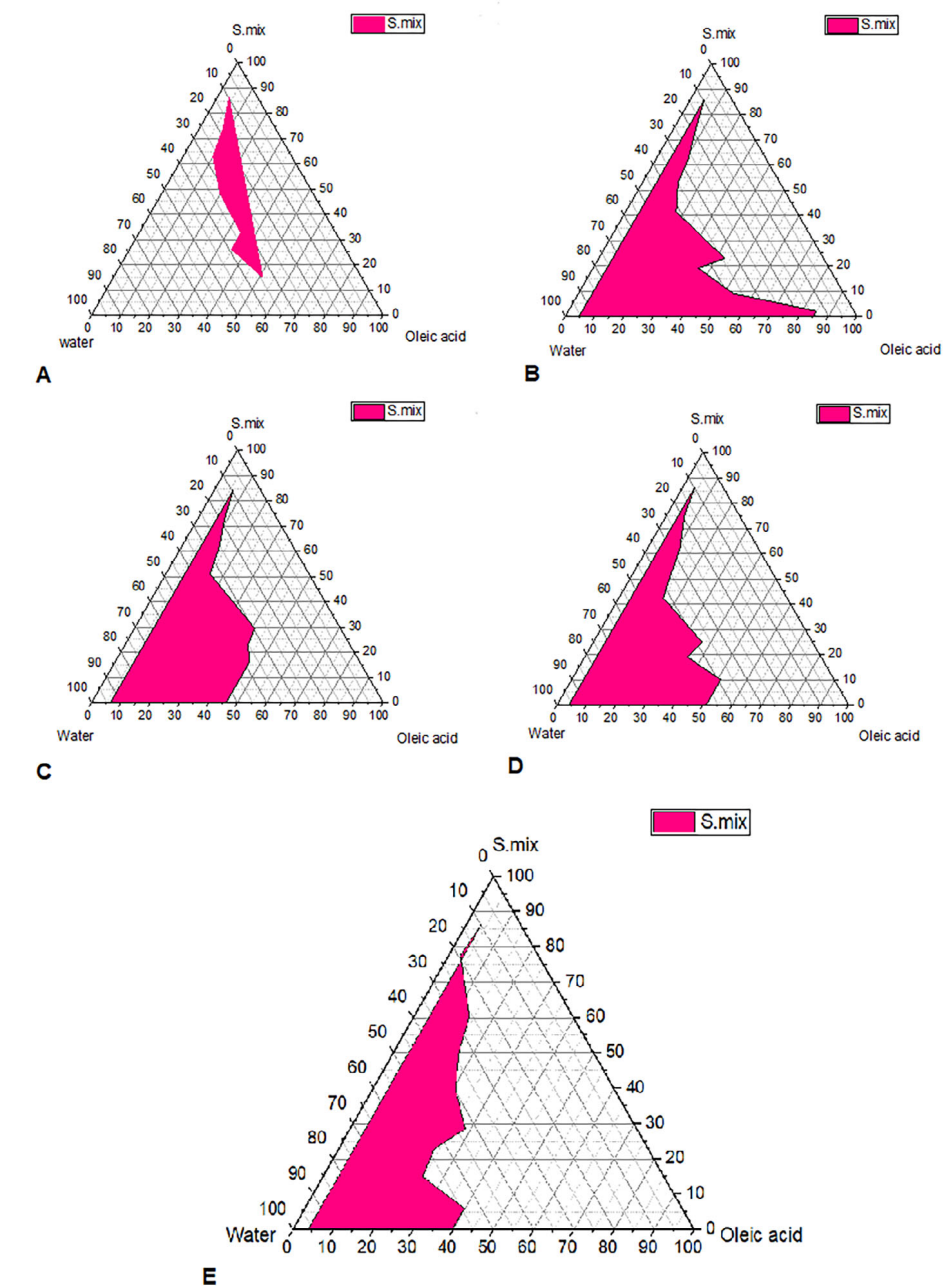
Pseudo ternary phase diagrams for self-microemulsion. Pseudo ternary phase diagrams for self-microemulsion composed of oil phase (Oleic acid), surfactant (Tween 80), cosurfactant (Transcutol P), cosolvent (propylene glycol) and water. (A) Smix 1:1, (B) Smix 1:2, (C) Smix 1:3, (D) Smix 1:4 and (E) Smix 3:4.

Pseudo-ternary phase diagrams were constructed to identify the self-emulsifying regions. The pseudo-ternary phase diagram were plotted for different Smix ratios (Tween 80: Transcutol P, Propylene glycol (1:1, 1:2,1:3,1:4 and 3:4)), as shown in Figure 1. ^45^ In the pseudo-ternary phase plot, the pink shaded area represents the area of microemulsions. The plot with a larger shaded area indicates the presence of good micro-emulsifying activity of formulated microemulsions and beneficial interaction among the Smix, oil and aqueous phase. Bancroft's rule states that the phase in which the surfactant is more soluble represents the continuous phase and determines the type of microemulsion produced (either o/w or w/o). ^25^ According to this rule, Tween 80 used as a surfactant, which is a hydrophilic molecule with HLB value of 15, is more soluble in aqueous phase and this favors the formation of o/w microemulsion. The pseudoternary phase diagrams demonstrate that the zone of microemulsion was largest in SMEDDS prepared with Tween 80:(Transcutol P, Propylene glycol Smix). Formulas selected with oil: Smix 1:9 ratios for all Smix ratios remained as microemulsions even upon infinite water titration or dilution. This is possible as Tween 80 with Transcutol P and propylene glycol mixture is strongly localized on the surface of the microemulsion droplets, minimizes the interfacial free energy and provides a mechanical barrier to coalescence resulting in a spontaneous dispersion. ^26^

Based these findings, the optimal micro-emulsification qualities are achieved with an increasing Tween 80 ratio. In order to avoid disagreeable side effects, it is crucial to accurately measure the surfactant concentration and use the optimal surfactant and co-surfactant concentrations in the formulation. ^27^

This current study involved the use of pre-concentrates consisting of oil and Smix, and the pseudoternary diagram was used to select the appropriate oil that solubilized the drug, surfactant and co-surfactant mixtures.

### Preparation of MLX liquid SMEDDS

There is no visible phase separation or drug precipitation in any of the observed liquid MLX SMEDDS formulations, and all of the mixtures are a uniform, clear and yellow to brown color.

***Assessment of the prepared liquid SMEDDS***

*Thermodynamic stability studies*

The thermodynamic stability test was passed by all of the prepared MLX SMEDDS formulations because there was no evidence of phase separation or drug precipitation at the end of all cycles. The purpose of this stability study is to identify metastable formulations and to suggest that the formulations were stable against storage in extreme conditions. ^28^

*Droplet size measurement and PDI*

The rate, extent, and absorption of drug release are all influenced by the droplet size of the microemulsion, making it the most important factor in self-emulsification performance. The relationship between droplet size and surface area explains the effect of microemulsion droplet size on drug release and bioavailability. It is well known that the smaller the droplet size, the greater the surface area available for drug release and absorption. ^29^

Table 5 ^45^ displays the droplet sizes and PDIs for the several SMEDDS formulations that were created. The droplet sizes ranged from 63.22 nm to 347.8 nm, and the PDIs ranged from 0.3 to 0.5069. The low polydispersity index indicates good droplet size distribution uniformity after dilution with water. ^30^ The results showed that the size of the droplets gets smaller as the Smix ratio and the ratio of surfactant to co-surfactant increase. This is because there are more surfactants at the oil/water interface. ^31^

**Table 5.** Droplet size measurements and PDI of meloxicam liquid SMEDDS. PDI, poly dispersity index; SMEDDS, Self-micro emulsifying drug delivery system.

F – code	Mean droplet size (in nm)	PDI
SMEDDS-1	347.8	0.435
SMEDDS-2	231.8	0.3766
SMEDDS-3	111.9	0.299
SMEDDS-4	158.7	0.5069
SMEDDS-5	63.22	0.3624

*Robustness to dilution*

Dilution is caused by gastrointestinal fluids, and it is impossible to precisely identify the amount of water present to form the microemulsion with the formulation. Dilution resistance was tested using an excess of water and 0.1 N HCl, and formulations were stored for 24 hours. This test was passed by all MLX SMEDDS formulations, which were visually examined as clear with no precipitation or phase separation. The ability of the SMEDDS formulation to withstand aqueous dilution was found to be excellent. This is due to the excipients' high solubilizing properties, as well as their ability to form a relatively stable microemulsion with small droplet sizes. This meant that the formulations were resistant to extreme dilution and could be diluted indefinitely with water. ^32^

*Dispersibility tests and self-nano emulsification time*

Emulsification studies are critical for determining the self-emulsifying properties of designed formulations. SMEDDS should completely and rapidly disperse in aqueous dilution with mild agitation. ^33^ When determining how effective emulsification is, one crucial index to consider is the rate at which it occurs. With grade A, all of the prepared MLX SMEDDS formulations formed the microemulsion in less than 1 minute. The difference in self-emulsification times between the different formulas in the bulk liquid SMEDDS was very small, and because the observation times were short (in seconds), it was difficult to distinguish between the formulas. Smix in SMEDDS reduces interfacial tension between the oil and aqueous phases, facilitating dispersion and the formation of o/w microemulsions. ^34^

*Drug content within the prepared SMEDDS*

Drug content of the prepared MLX SMEDDS at the nanoscale was more than 97%, which meets USP requirements and is within an acceptable range (90–110%), indicating that there was no precipitation of drug in any of the prepared formulations. ^35^ The drug content percentage of the MLX SMEDDS is shown in Table 6. ^41^

**Table 6.** Drug content percentage of meloxicam liquid self-microemulsion. Data are presented as mean±SD, n=3. SMEDDS, Self-micro emulsifying drug delivery system.

F – code	Drug content %
SMEDDS-1	97.65±0.05
SMEDDS-2	97.596±0.189
SMEDDS-3	99.463±0.116
SMEDDS-4	98.423±0.266
SMEDDS-5	99.653±0.151

*In vitro dissolution study*

**Figure 2.**
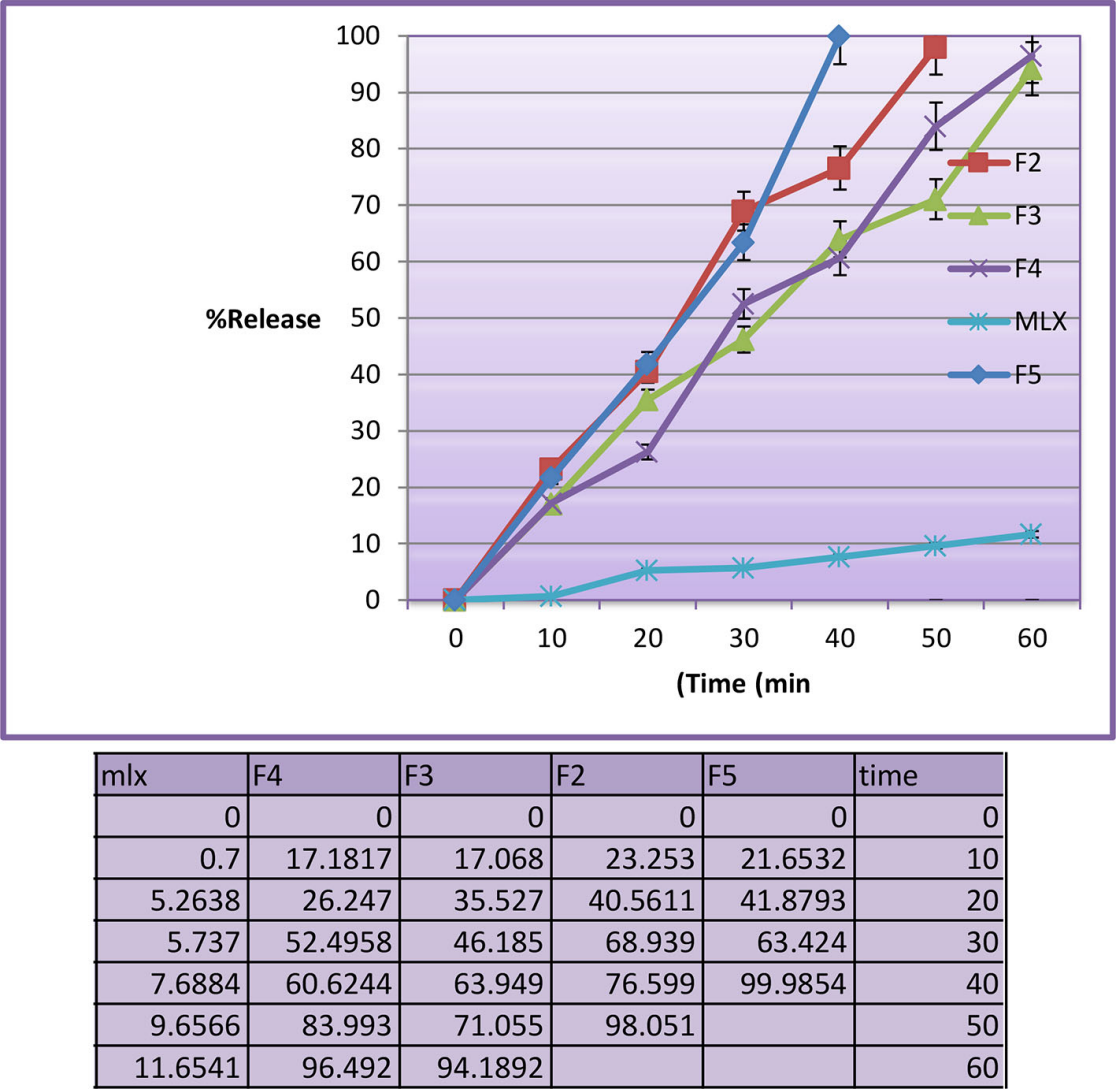
Dissolution profile of MLX SMEDDS (F2, F3, F4 and F5) and pure meloxicam. MLX, meloxicam; SMEDDS, Self-micro emulsifying drug delivery system.

The *in vitro* drug release profiles regarding F2 to F5 and pure MLX have been assessed in 0.1 N HCl, 50 rpm and 37°C are shown in Figure 2. ^43^ The F1 was disregarded due to the size of the droplet (347.8 nm). Dialysis membranes were used in this test because they are less prone to blockage and have very small pores. ^5^ The prepared MLX SMEDDS formulations showed drug release percentage of more than 94% at the end of 60 min. However the F5 showed higher release percentage of 99.87% at 40 min. The drug is released more quickly because it is dissolved in the SMEDDS. The faster release is due to the fine particle size and high concentration of surfactant mixture, which can easily emulsify the oil for finer globules. ^36^

Pure MLX has a slower release profile than prepared SMEDDS formulations, reaching 11.6% after 60 minutes in the absence of a dialysis membrane. The release profile of the ordinary MLX powder is significantly different from the prepared SMEDDS formulations. Finally, the SMEDDS formulations resulted in the spontaneous creation of microemulsion with tiny droplet size, which enabled rapid rate of the drug release to the aqueous phase, much quicker than that of the pure drug powder. F5 has significant differences from other formulations (similarity factor<50).

### Selection of optimum MLX liquid self-microemulsion

F5 was chosen as the optimal MLX liquid self-microemulsion due to its higher drug content, greater *in vitro* release, smaller droplet size, and lower PDI value.

***Zeta potential measurement***

**Figure 3.**
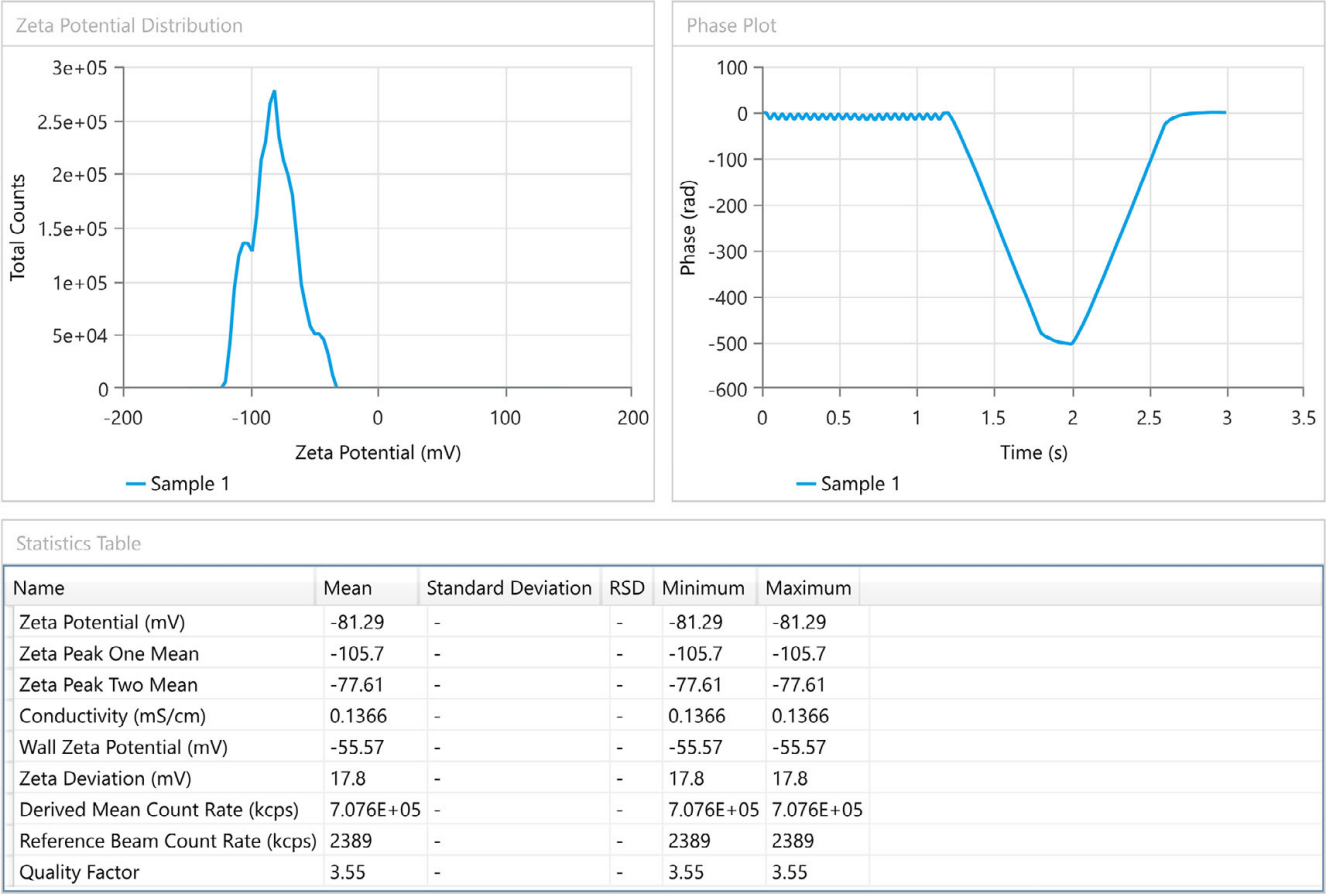
Zeta potential of SMEDDS–F5. SMEDDS, Self-micro emulsifying drug delivery system.

The zeta potential of an emulsion reveals how much force is exerted by the droplets against one another. No matter their charge, a normal zeta potential value is above 30 mV, ^37^ for just accepted absolute electrostatic stabilization. Zeta potential value of the produced microemulsion was found to be -81.29 mV, as shown in Figure 3. ^46^ Anionic groups of fatty acids and glycols in oils, surfactants, and co-surfactants may contribute to the occurrence of negative zeta potential. This test was performed only for the selected formula F5.

The droplets' negative charge would prevent them from clumping together. Emulsifiers prevent oil droplets from coalescing in part by acting as a mechanical barrier and in part by forming surface charges (zeta potential) that can produce repulsive electrical forces between approaching oil droplets.

Due to the enhanced zeta potential (negative charge) and steric stabilization effect, the optimized SMEDDS (F5) does not display threshold agglomeration. ^38^

***Atomic force microscopy (AFM)***

**Figure 4.**
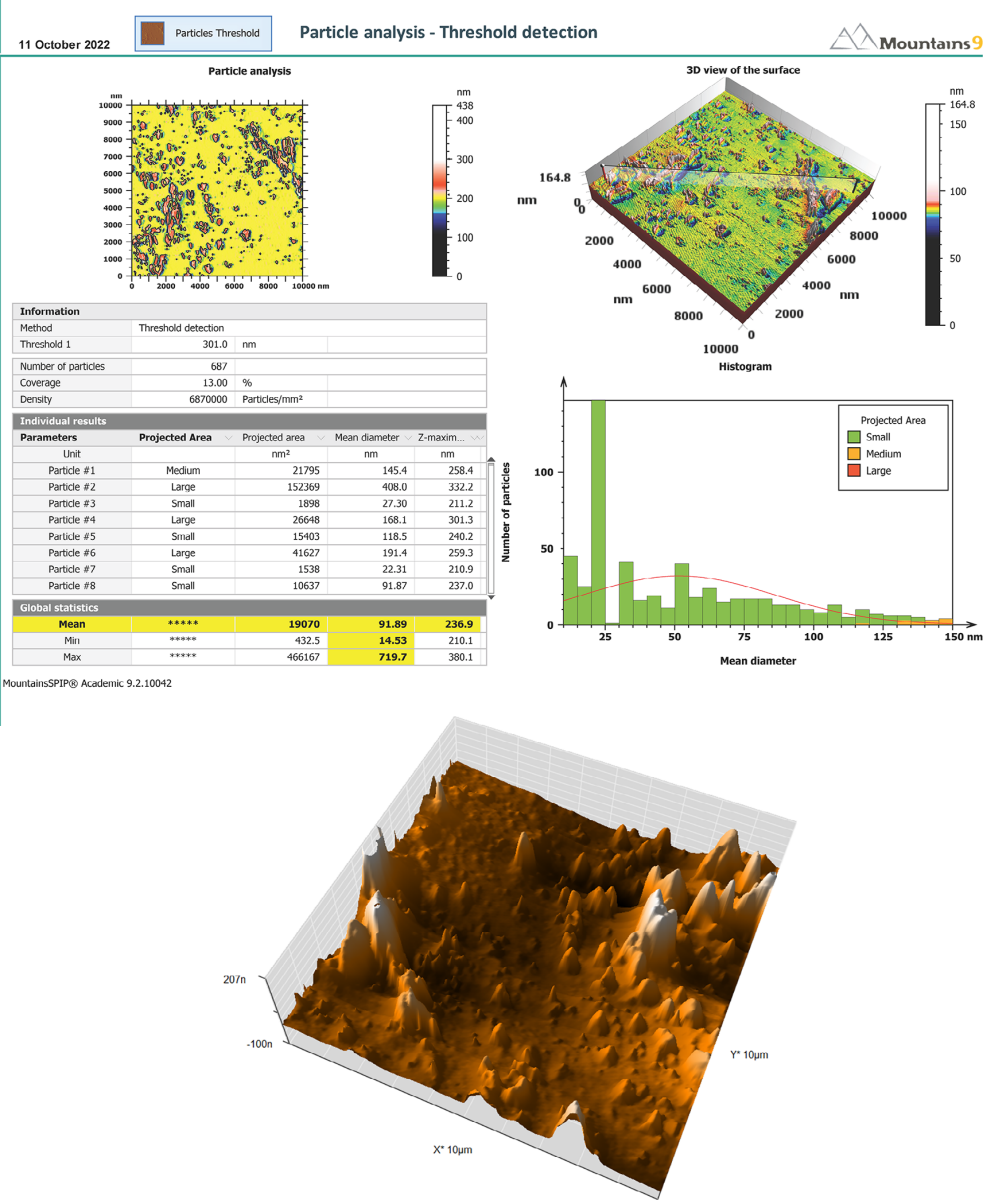
AFM of SMEDDS-F5. (A) Report of AFM. (B) 3D-surface morphology. AFM, Atomic force microscopy; SMEDDS, Self-micro emulsifying drug delivery system.

In addition to proving the nanoscaled potential of microemulsions by conventional means, AFM topographic pictures give a broad size summary and defining the form and surface structure of the studied sample. ^39^ The mean size of the droplet was 91 nm with smooth surface of the formula. The AFM results are shown in Figure 4.

***Field emission scanning electron microscopy (FESEM*** **)**

**Figure 5.**
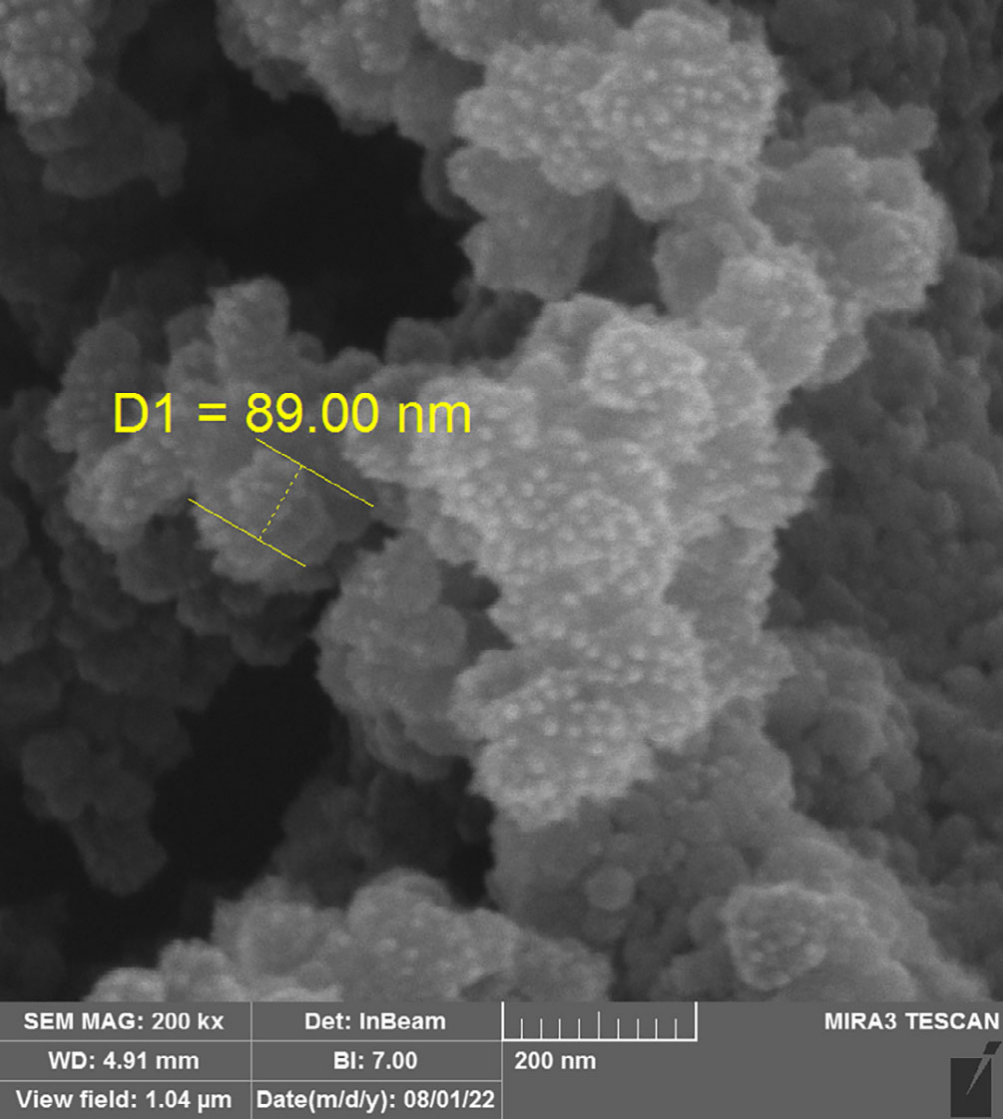
FESEM image of MLX selected F5. FESEM, Field emission scanning electron microscope; MLX, meloxicam.

The result shows a droplet size of 89 nm that is around AFM result, which means that the formula is monodispersed and the shape of the droplet is spherical. The result of FESEM is shown in Figure 5.

***Transmission electron microscopy (TEM)***

**Figure 6.**
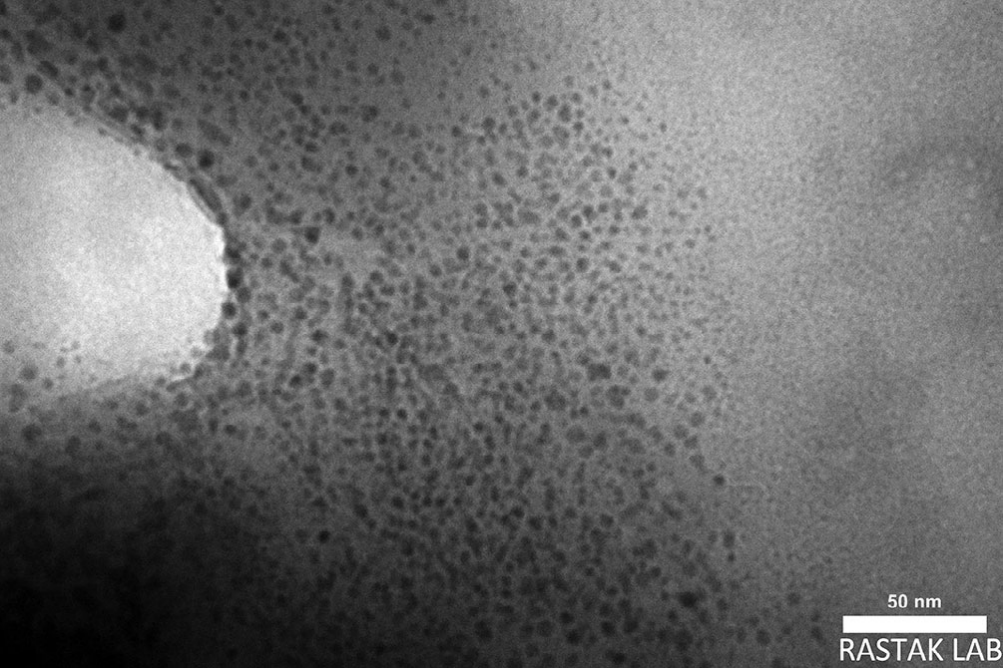
TEM of SMEDDS-F5. TEM, Transmission Electron Microscope; SMEDDS, Self-micro emulsifying drug delivery system.

TEM defines the morphology of microemulsion, as seen in Figure 6, spherical shape and uniform nanometric size of the droplets with smooth surface of the formula.

## Conclusions

The results of this research indicate that SMEDDS is a promising route for developing an oral dosage regimen for MLX that is poorly soluble in water. The SMEDDS method was crucial in enhancing the stability, hydrophilicity, dissolution, and dissolution rates of MLX. The F-5 (10% oleic acid oil, 38.57% Tween 80, 25.72% propylene glycol, and 25.72% Transcutol P) showed a higher release percentage of 99.87% at 40 minutes. The drug is released more quickly because it is dissolved in SMEDDS. The faster release is due to the large surface area due to the fine droplet size and high concentration of the surfactant mixture, which can easily emulsify the oil into finer globules. The best formula has a zeta potential of -81 mV, which means it is stable. The AFM, FESEM, and TEM showed uniform particle size at the nanoscale and a spherical particle shape with a smooth surface. Drug solubility is enhanced by nanosized formulations because of the greater surface area provided for drug release and absorption.

## Data Availability

Zenodo: Cosurfactants saturation solubility. https://doi.org/10.5281/zenodo.7600515 ^40^ Zenodo: drug cotents. https://doi.org/10.5281/zenodo.7600527 ^41^ Zenodo: Oils saturation solubility. https://doi.org/10.5281/zenodo.7600535 ^42^ Zenodo: RELEASE OF FORMULATIONS. https://doi.org/10.5281/zenodo.7600539 ^43^ Zenodo: Surfactants saturation solubility. https://doi.org/10.5281/zenodo.7600545 ^44^ Zenodo: Formulation size and ternary plot. https://doi.org/10.5281/zenodo.7600547 ^45^ Zenodo: Image for (Formulation and in vitro evaluation of meloxicam as a self-microemulsifying drug delivery system). https://doi.org/10.5281/zenodo.7660921 ^46^ Data are available under the terms of the Creative Commons Attribution 4.0 International license (CC-BY 4.0).
