# Supplementary material for: Formulation and in vitro evaluation of meloxicam as a self-microemulsifying drug delivery system
Source: F1000Res. 2023 May 30;12:315. Originally published 2023 Mar 22. [Version 2] doi: 10.12688/f1000research.130749.2 (PMC10285354; doi:10.12688/f1000research.130749.2)
Supplement: Peer review history

# Peer review: Reviewer response for version 2

**DOI:** 10.5256/f1000research.148205.r175873

**Published:** 2023-06-12

**Authors:** Mohd Yasir

**Affiliations:** 1 Department of Pharmacy, College of Health Science, Arsi University, Assela, Oromia, Ethiopia

Thank you for providing me with an opportunity to re-review the said manuscript entitled “Meloxicam as a self-microemulsifying drug delivery system for oral delivery” The author addressed almost all the comments provided by me last time.  At present, the manuscript is looking better. Now I am in a position to give my final suggestion to approve the manuscript.

Is the work clearly and accurately presented and does it cite the current literature?

Yes

If applicable, is the statistical analysis and its interpretation appropriate?

Yes

Are all the source data underlying the results available to ensure full reproducibility?

Yes

Is the study design appropriate and is the work technically sound?

Yes

Are the conclusions drawn adequately supported by the results?

No

Are sufficient details of methods and analysis provided to allow replication by others?

Yes

Reviewer Expertise:

Pharmaceutics (Formulation design, Nano drug delivery, Nose to brain drug delivery, Transdermal drug delivery, Oral drug delivery, Lipid Naoparticles, etc

I confirm that I have read this submission and believe that I have an appropriate level of expertise to confirm that it is of an acceptable scientific standard.

# Peer review: Reviewer response for version 2

**DOI:** 10.5256/f1000research.148205.r175874

**Published:** 2023-06-06

**Authors:** Michael Adu-Frimpong

**Affiliations:** 1 Department of Applied Chemistry and Biochemistry, Faculty of Applied Sciences, C. K. Tedam University of Technology and Applied Sciences (CKT-UTAS), Navrongo, Ghana

The manuscript can be indexed in this form

Is the work clearly and accurately presented and does it cite the current literature?

Yes

If applicable, is the statistical analysis and its interpretation appropriate?

Yes

Are all the source data underlying the results available to ensure full reproducibility?

Yes

Is the study design appropriate and is the work technically sound?

Yes

Are the conclusions drawn adequately supported by the results?

Yes

Are sufficient details of methods and analysis provided to allow replication by others?

Yes

Reviewer Expertise:

Preparation of delivery systems for active compounds of plants

I confirm that I have read this submission and believe that I have an appropriate level of expertise to confirm that it is of an acceptable scientific standard.

# Peer review: Reviewer response for version 1

**DOI:** 10.5256/f1000research.143527.r169588

**Published:** 2023-04-18

**Authors:** Michael Adu-Frimpong

**Affiliations:** 1 Department of Applied Chemistry and Biochemistry, Faculty of Applied Sciences, C. K. Tedam University of Technology and Applied Sciences (CKT-UTAS), Navrongo, Ghana

**Reviewer’s comment**

 Muhammad et al incorporated meloxicam (MLX) into self-microemulsifying drug delivery system, which improved solubility and oral bioavailability of the drug. This work is relevant for development of drug delivery system. The authors need to address the following concerns before possible indexing.

**General comment**

 The particle size of the formulation is in nanoscale. Why the authors did use ‘microemulsifying’ instead of ‘nanoemulsifying’   

**Introduction**

1. In order to justify the need for this delivery system for MLX, the authors should indicate other existing delivery systems of the drug and their shortcomings

**Methods and materials**

1. In determining the drug content, did authors estimate of the entrapment efficiency and loading capacity of MLX liquid SMEDDS?
2. Why did the authors determine in vitro drug release in only pH 1.2 but not in pH 7.4?

**Results and Discussion**

1. Based on these findings, the optimal micro-emulsification qualities... seems better
2. In confirming the transparency of the formulation, did the authors determine the percentage transmittance of the MLX liquid SMEDDS?
3. The authors should explain why the SMEDDS formulations resulted in the spontaneous creation of microemulsion with tiny droplet size
4. The authors should comment on the stability of the formulation. Did the authors study the long-term storage stability of MLX liquid SMEDDS?

Is the work clearly and accurately presented and does it cite the current literature?

Yes

If applicable, is the statistical analysis and its interpretation appropriate?

Yes

Are all the source data underlying the results available to ensure full reproducibility?

Yes

Is the study design appropriate and is the work technically sound?

Yes

Are the conclusions drawn adequately supported by the results?

Yes

Are sufficient details of methods and analysis provided to allow replication by others?

Yes

Reviewer Expertise:

Preparation of delivery systems for active compounds of plants

I confirm that I have read this submission and believe that I have an appropriate level of expertise to confirm that it is of an acceptable scientific standard, however I have significant reservations, as outlined above.

# Response

**Published:** 2023-05-12

**Authors:** Saja Muhammed

**Affiliations:** Department of Pharmaceutics, College of Pharmacy, University of Baghdad, Iraq

**Thanks for your response, your time, your rich information, and your helpful** **review.**

**I will reply to all comments below.**

**General comment**

**The particle size of the formulation is in nanoscale. Why the authors did use ‘microemulsifying’ instead of ‘nanoemulsifying’**

 Knowing that microemulsion droplet size is in the nanoscale and not in the microscale which is misleading most of the times but it is a known fact.

**Introduction**

**In order to justify the need for this delivery system for MLX, the authors should indicate other existing delivery systems of the drug and their shortcomings**

 There are many delivery systems that can be used to formulate MLX, like nanoemulsion, microemulsion, soild dispersion, etc.

 The microemulsion delivery system has the disadvantage of having a large amount of surfactants; stability is influenced by temperature and pH; and substances with high melting points have limited solubility capacity.

 The nanoemulsion delivery system has the disadvantage of a large concentration of the surfactant and expensive techniques for preparation.

 The disadvantages of solid dispersion are that they form crystalline solid dispersion as they are prepared using crystalline carriers like urea and sugars. Crystalline solid dispersions were more thermodynamically stable, which lowered their dissolution rate as compared to amorphous ones.

 While the SMEDDS deliver the drug with the easiest technique and faster drug release.

**Methods and materials**

**In determining the drug content, did authors estimate of the entrapment efficiency and loading capacity of MLX liquid SMEDDS** **?**

 I respect your opinion but since the system is not vascular most of the reference not include this test and only carried out the drug contents which is give a good result of drug loading.

**Why did the authors determine in vitro drug release in only pH 1.2 but not in pH 7.4**

 SMEEDS delivery system is not conventional dosage form,and we know that meloxicam is a weakly acidic drug and release is fater in the acidic media,and this article is a part of thesis in which we actually use 7.4 PH buffer which give slower release than 1.2 PH media.

**Based on these findings, the optimal micro-emulsification qualities... seems better**

****

**In confirming the transparency of the formulation, did the authors determine the percentage transmittance of the MLX liquid SMEDDS**

 The most of references in our study don’t determine the percentage transmittance of the liquid SMEDDS, they did this test mostly  in micro and nano emulsion and also this system is not vascular system and the formula is a clear liquid as you noticed in the results.

**The authors should explain why the SMEDDS formulations resulted in the spontaneous creation of microemulsion with tiny droplet size**

 Due to interfacial tension caused by the s.mix which resulted in tiny droplets with different nanoscales.

**The authors should comment on the stability of the formulation. Did the authors study the long-term storage stability of MLX liquid SMEDDS?**

 Due to highly thermodynamic stability of the formulations and this article is a part of  thesis that study the long term stability of the formulations for three months and give a good result of release and drug content.

# Peer review: Reviewer response for version 1

**DOI:** 10.5256/f1000research.143527.r167515

**Published:** 2023-04-11

**Authors:** Mohd Yasir

**Affiliations:** 1 Department of Pharmacy, College of Health Science, Arsi University, Assela, Oromia, Ethiopia

Dear Author

 Thank you for providing a study associated with “Meloxicam as a selfmicroemulsifying drug delivery system for oral delivery”. The author requires to respond to the following major comments before the final decision on the manuscript  

**Comments **

1. Author is required to mention physiochemical characters that make Meloxicam as a suitable drug for designing  SMEDDS. 
2. Please mention the specifications (model number, company, etc)  of all machines like centrifuge, used for the research.
3. During the mixing for preparation of SMEDDS/Smix, what was the end point of mixing of oil with Trizma buffer (water), etc. how they identify the final end point.
4. For the measurement of droplet size  and polydispersity index, dilution was made with distilled water but in my opinion it should made with deionized water.
5. Druing measurement of drug content, what was the value of absorption maxima (ʎmax) used for UV/visible Spectrophotometer measurement. Moreover Please mentioned the model number and company of UV/visible Spectrophotometer in bracket.
6. Better if author describe the zeta potential after particle size. So move it from here and describe just after particle & PDI heading in both cases viz method and result & discussion.
7. Page 8: Author mentioned  the reference in Table 5 ^45^  . Have they taken this table from this reference.  Please check it
8. Why the author gave the reference in this sentence “The drug content percentage of the MLX SMEDDS is shown in Table 6. ^41^”. Please check it and correct.
9. Again same issue associated reference  “The in vitro drug release profiles regarding F2 to F5 and  pureMLXhave been assessed in 0.1NHCl, 50 rpm and 37°C are shown in Figure 2. ^43^”. please check it and remove the reference.
10. Please add the error bars in figure 2
11. Please elaborate the conclusion
12. Check the manuscript for the grammar and punctuation issue.

Is the work clearly and accurately presented and does it cite the current literature?

Yes

If applicable, is the statistical analysis and its interpretation appropriate?

Yes

Are all the source data underlying the results available to ensure full reproducibility?

Yes

Is the study design appropriate and is the work technically sound?

Yes

Are the conclusions drawn adequately supported by the results?

No

Are sufficient details of methods and analysis provided to allow replication by others?

Yes

Reviewer Expertise:

Pharmaceutics (Formulation design, Nano drug delivery, Nose to brain drug delivery, Transdermal drug delivery, Oral drug delivery, Lipid Naoparticles, etc

I confirm that I have read this submission and believe that I have an appropriate level of expertise to confirm that it is of an acceptable scientific standard, however I have significant reservations, as outlined above.

# Response

**Published:** 2023-05-12

**Authors:** Saja Muhammed

**Affiliations:** Department of Pharmaceutics, College of Pharmacy, University of Baghdad, Iraq

**Thanks for your response, your time, your rich information, and your helpful** **review.**

**I will reply to all comments below.**

**Comments**

**Author is required to mention physiochemical characters that make Meloxicam as a suitable drug for designing  SMEDDS**

 Meloxicam is suitable for this system due to its poor water solubility, which causes low absorption, low dosage, no substantial first-pass metabolism, is highly soluble in the system ingredients, and has a high log p of 3.43This is added to the article

**Please mention the specifications (model number, company, etc)  of all machines like centrifuge, used for the research**

 Centrifuge,

 Eppendorf, Germany,

 Hettich EBA20

**During the mixing for preparation of SMEDDS/Smix, what was the end point of mixing of oil with Trizma buffer (water), etc. how they identify the final end point**

 In the preparation of the SMEDDS the mixing of ingredients according to specific weight of them and mixing until obtain a clear liquid.

**For the measurement of droplet size  and polydispersity index, dilution was made with distilled water but in my opinion it should made with deionized water.**

 I respect your opinion but the instrument operator usually use a double distilled water in our measurements.

**During measurement of drug content, what was the value of absorption maxima (ʎmax) used for UV/visible Spectrophotometer measurement. Moreover Please mentioned the model number and company of UV/visible Spectrophotometer in bracket**

 The value of absorption maxima (ʎmax) used for UV/visible Spectrophotometer measurement is 355 nm

 UV-Visible Spectrophotometer

 Shimadzu 1650 pc-Japan

 UV mini 1240

 This is added to the article.

**Better if author describe the zeta potential after particle size. So move it from here and describe just after particle & PDI heading in both cases viz method and result & discussion.**

 I agree with your opinion but since the zeta potential is done on the optimum formula so we put it according  to the sequence of the steps in the study

**Page 8: Author mentioned  the reference in Table 545  . Have they taken this table from this reference.  Please check it**

****

**Why the author gave the reference in this sentence “The drug content percentage of the MLX SMEDDS is shown in Table 6.41”. Please check it and correct**

****

**Again same issue associated reference  “The in vitro drug release profiles regarding F2 to F5 and  pureMLXhave been assessed in 0.1NHCl, 50 rpm and 37°C are shown in Figure 2.43”. please check it and remove the reference**

 Those tables are the result of the study but due to the guidelines of the journal this reference is only to get the table in more details with the underlying data.

**Please add the error bars in figure 2**

 See amended figure here. This also added in the article's figure

**Please elaborate the conclusion**

 The results of this research indicate that SMEDDS is a promising route for developing an oral dosage regimen for MLX that is poorly soluble in water. The SMEDDS method was crucial in enhancing the stability, hydrophilicity, dissolution, and dissolution rates of MLX. The F-5 (10% oleic acid oil, 38.57% Tween 80, 25.72% propylene glycol, and 25.72% Transcutol P) showed a higher release percentage of 99.87% at 40 minutes. The drug is released more quickly because it is dissolved in SMEDDS. The faster release is due to the large surface area due to the fine droplet size and high concentration of the surfactant mixture, which can easily emulsify the oil into finer globules. The best formula has a zeta potential of -81 mV, which means it is stable. The AFM, FESEM, and TEM showed uniform particle size at the nanoscale and a spherical particle shape with a smooth surface. Drug solubility is enhanced by nanosized formulations because of the greater surface area provided for drug release and absorption.

 This is added in the article.
